# Elevated LV Mass and LV Mass Index Sign on the Athlete’s ECG: Athletes’ Hearts are Prone to Ventricular Arrhythmia

**DOI:** 10.3390/jcm7060122

**Published:** 2018-05-28

**Authors:** Mücahid Yılmaz, Hidayet Kayançiçek

**Affiliations:** 1 Department of Cardiology, Elazığ Education and Research Hospital, Elazığ-23200, Turkey; 2 Department of Cardiology, Elazığ Medical Park Hospital (Affiliated to Istinye University), Elazığ-23040, Turkey; dr.hidayet@hotmail.com

**Keywords:** athlete’s heart, left ventricular mass, left ventricular mass index, sudden cardiac death, arrhythmia

## Abstract

Objectives: Intense exercise elevates all heart chambers’ dimensions, left ventricular mass (LV mass), and left ventricular mass index (LV mass index). The relationship between increased ventricular arrhythmias and sudden cardiac death with LV dilatation and elevated LV mass has been previously demonstrated. We investigated whether sports-related LV dilatation and elevated LV mass and LV mass index cause an increase in ventricular repolarization heterogeneity. Patients and Methods: This prospective observational study recruited 565 participants. There were 226 (female: 28) athletes and 339 (female: 45) healthy controls between 17 and 42 years of age. They were evaluated using 12-lead-electrocardiography and transthoracic echocardiography. Electrocardiograms were obtained at a rate of 50 mm/s and an amplitude of 10 mV, including at least 3 QRS complexes for each derivation. They were taken with 12 standard deviations. Transmural dispersion of repolarization indexes (TDR) (Tp-Te interval, Tp-Te/QT ratio and Tp-Te/QTc ratio, Tp-Te(d)) were measured from precordial derivations. Measurements weretakenwith a program which was generated with MATLAB codes. Results: Tp-Te interval, Tp-Te/QT ratio, Tp-Te/QTc ratio, Tp-Te(d), PW (posterior wall thickness), IVS (interventricular septal thickness), LVEDD (left ventricular end-diastolic diameter), LV mass (left ventricular mass), and LV mass index (left ventricular mass index) for the athlete group were significantly higher than for the control group. Correlation analyses revealed that TDR indexes significantly correlated with PW, IVS, LVEDD, LV mass, and LV mass index. Conclusion: LV mass and LV mass index increase in well-trained athletes, and this increase leads to an increase in TDR indexes. The increased frequency of ventricular arrhythmia and sudden cardiac death may be explained with increasing ventricular repolarization heterogeneity in these individuals.

## 1. Introduction

The heart of an athlete who does long-term training and has normal physiological and morphological changes is known as athlete’s heart syndrome, athletic heart syndrome or athletic heart. This adaptation is accepted as a normal physiological response of the heart to repetitive exercise [1]. It includes a decrease in the resting heart rate and an increase in the left ventricle mass and volume although the systolic and diastolic functions are normal. These are the features of athletic heart [2]. The level of the changes that occur in the heart varies according to the type of sport. Increases in the left ventricle mass and dilatation are seen in void diameters (eccentric hypertrophy) in sports that require high endurance (e.g., football). In sports that require high-level force (e.g., weight lifting), however, an increase is observed in the left ventricle mass, although the diameters of voids do not change (concentric hypertrophy). In athletes participating in sports that require high endurance and high force (e.g., rowing), eccentric and concentric hypertrophy occur together [3]. Although ischemia may develop and cause malignant arrhythmia in athletes for whom such sports cause abnormal ventricular hypertrophy, it is questionable whether the hypertrophy that occurs as a physiological response in healthy individuals may cause such a risk. Another hypothesis suggests that the action potential time in myocardia cells that occurs as a result of hypertrophy causes the elongation of the repolarization time, which might prepare the ground for ventricular arrhythmia [1,4].

Myocardia repolarization time is associated with being sensitive to ventricular tachyarrhythmia mainly with *torsades de pointes*. This may transform into ventricular fibrillation [5]. These measurements may be made by computing the Tp-e interval, which is accepted as the indicator of QT interval (QT), QT dispersion (QTd) and ventricle repolarization total dispersion (transmural, apicobasaland global) [6,7,8]. In a recent study conducted by Castro Hevia J et al. [9], it was reported that there is a close relation between life-threatening ventricular arrhythmia and the elongation of these parameters. This study used Tp-e dispersion (Tp-e) d values and elongated Tp-e interval in patients with Brugada Syndrome [9]. It has been shown in recent studies that the Tp-e/QT ratio provides accurate predictive values for ventricular arrhythmia and might be used as a novel ventricular arrhythmia index [10,11].

Sudden death seen in athletes has caused societal concern about this topic. The post workout death of professional footballer, Davide Astori, on 4 March 2018 renewed public interest in the deaths of athletes. He was the captain of Fiorentina FC. Do athletes with athletic heart have a greater risk for sudden death than healthy non athletes? Is the athlete’s heart proarrhythmic? Are there electrocardiographic (ECG) data that might reveal the tendency of athletic heart to have arrhythmia? It has not yet been shown whether the physiological changes observed in professional athletes lay the foundation for ventricle repolarization changes, and thus, for malignant ventricular arrhythmia. Many studies have been conducted on athletes to evaluate the ventricle repolarization QT time with parameters, such as QT dispersion [2,12,13,14]. The purpose of this study is to determine the potential increased ventricle repolarization heterogeneity of athletes and possible arrhythmia risks which may develop due to intense training in asymptomatic athletes. In this context, we sought to contribute to the ongoing discussions on the athletic heart–arrhythmia axis by using the computations of Tp-Te time, Tp-Te/QT ratio, Tp-Te/QTc ratio, and Tp-Te dispersion Tp-Te(d) through 12-derivation surface electrocardiogram (ECG) in athletes.

## 2. Materials and Method

In this study, 226 well-trained athletes or well-trained students from Fırat University, Physical Education and Sports High School were admitted to the outpatient clinics of Elazıg Education and Research Hospital, Elazıg, Turkey between July 2016 and February 2018.They received routine annual follow-ups or licenses. A total of 339 healthy controls were also included in the study. The participants’ ages ranged from 17 to 42 years. The cardiovascular system and other system examinations of the participants were normal, and they did not have any known systemic diseases. No participants had any complaints that could be associated with dysrhythmia, such as palpitations or fainting. There were no histories of sudden death or cardiovascular disease that could cause sudden death. The average training duration was 14.15 ± 6.57 years in the athlete group. The weekly training durations of the athletes were 8.67 ± 1.97 hours. They trained once a day. The daily training programs were in the form of 30-min isometric exercise, and then 60-min to 120-min athletic exercises, boxing, kick-boxing, fitness, handball, football, and basketball.

The study was performed in accordance with Helsinki principles and approved by the local Ethics Committee (Presidency of T.C. Fırat University Ethics Committee).

### 2.1. Echocardiography

Transthoracic echocardiography was performed using a Vivid 5 instrument (GE Medical Systems, Milwaukee, WI, USA) with a 2.5 MHz transducer and harmonic imaging according to the recommendations of the American Society of Echocardiography [15]. Left ventricular systolic and diastolic diameters were measured with M-mode echocardiography. The left ventricular mass (LVM) was calculated according to a previously published methodology [16]. LVM (g) = 0.8{1.04[([LVEDD + IVSd + PWd]^3^ − LVEDD^3^)]} + 0.6, where IVST is interventricular septum thickness, LVEDD is left ventricular end-diastolic diameter and PWT is posterior wall thickness. LVMI (g/m^2^) was calculated as follows: LVMI = Left ventricular mass/body surface area [16]. Relative wall thickness (RWT) allows for further classification of LV mass increases as either concentric hypertrophy (RWT > 0.42) or eccentric hypertrophy (RWT ≤ 0.42). RWTwas obtained as follows: RWT = 2 × PWT/LVEDD [16]. LV end-diastolic volume (LVEDV) and LV end-systolic volume (LVESV) were calculated as LVEDV = (7/2.4 + LVEDD) × (LVEDD^3^) and LVESV = (7/2.4 + LVESV) × (LVESV^3^), respectively [17]. LVEDVindex (LVEDVI) was calculated as follows: LVEDVI (mL/m^2^) = LVEDV/body surface area (BSA) [18]. Ejection fraction (EF) was determined as: EF = (LVEDV − LVESV)/(LVEDV × 100) [16]. Fractional shortening (FS) was calculated by the following equation: FS = (LVEDV − LVESV)/(LVESV × 100) [17].

### 2.2. Electrocardiography

**Figure 1:**
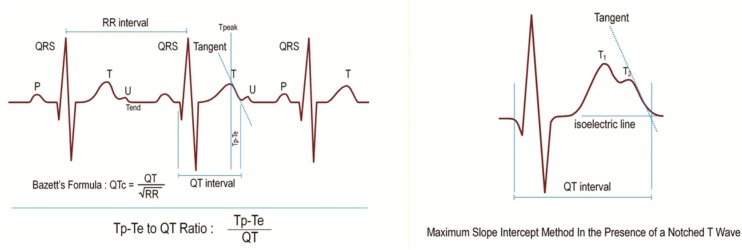
T wave peak-to-end interval (Tp-Te), QT interval, RR interval, Bazzett’s Formula, Tp-e/QT Ratio, and maximum slope intercept method in the presence of a notched T wave.

The 12-lead ECG recordings were obtained while the participants were in the supine position. There was a paper speed of 50 mm/s and a voltage of 10 mm/mV with the standard ECG system (Cardiofax V model 9320, Nihon Kohden, Tokyo, Japan). All ECGs were scanned. The T wave peak-to-end interval (Tp-Te) and QT and RR intervals were measured using MATLAB^®^ computer software (MathWorks, Natick, MA, USA) codes that were written by an engineer (Figure 1). These codes were based on image manipulation principles.

The image manipulation method can be divided into three subdivisions: image processing, image analysis, and image understanding. Image analysis is the technique used to gather measurement data from the ECG. Running the written code imports the image file first and then, by choice, allows the user to choose points necessary to obtain measurements or generate a matrix that consists of a dedicated numeric value of each pixel’s colour. Creating a matrix gives the user the flexibility of using functions which are predefined by the program.

The QT interval was defined from the beginning of the QRS to the end of the T wave as the intersection of the tangent to the downslope of the T wave and the isoelectric line [19]. Measurements of the QT interval were recorded from all precordial leads. The greatest values were accepted as the QTmax. The QTc (QT corrected) was obtained using Bazett’s formula [20] (Figure 1). The Tp-Te interval was defined as the interval from the peak of the T wave to the end of the T wave (Figure 1). With negative or biphasic T waves, the T peak was defined as the nadir of the Twave. In the presence of a notched T wave, the QT interval was determined using the tangent line drawn on top of the interception point of the start of QRS and the isoelectric line and the maximum slope of the second notch (Figure 1). T waves smaller than 1.5 mm in amplitude were not measured. Measurements of Tp-Te interval were recorded from all precordial leads. The greatest values were accepted as T wave peak-to-end intervals (Tp-Te). T wave peak-to-end interval dispersion Tp-Te(d) was calculated as the difference between the maximum and minimum Tp-Te interval in the precordial leads V1 to V6. The Tp-Te/QT ratio was calculated using Tp-Te and QTmax measurements. The Tp-Te/QTc ratio was obtained by dividing Tp-Te interval by the QTc.

### 2.3. Exclusion Criteria of the Study

Parameters, such as body mass index (BMI), body surface area (BSA), and blood pressure, were measured with the physical examination. BMI and BSA were calculated as indicated in the formulas below.

Body mass index: Weight (kg)/height (m) and Mosteller Formula: BSA (m^2^) = (height (cm) × weight (kg)/3600)^1/2^.

The blood pressure records of the participants were noted. The participants having a systolic blood pressure ≥140 mmHg and/or a diastolic blood pressure ≥90 mmHg and those taking antihypertensive drugs were accepted as hypertensive and excluded from the study.

Venous blood samples were collected from each participant in order to assess serum biochemical parameters. The participants having measurements of fasting blood glucose levels ≥126 mg/dL or using oral antidiabetic drugs or insulin were accepted as diabetic and excluded from the study.

The participants who had right or left bundle-branch block in 12-derivation surface ECG, had atrial or ventricular arrhythmia, used antiarrhythmic agents that might cause arrhythmia (such as probucol, terfenadine, amiodarone, erythromycin, clarithromycin, antidepressant agents, and antipsychoticagents), had electrolyte disorder, had BMIs >30, had structural heart disease, and were pregnant were not included in the study.

### 2.4. Statistical Evaluation

Statistical analyses were performed using SPSS software, version 16.0 (SPSS Inc., Chicago, IL, USA) for Windows. Continuous variables were expressed as mean standard deviations and categorical variables were expressed as counts and percentages. The student *t*-test and the Mann–Whitney U test were used to compare groups for continuous variables. The chi square test was used for categorical variables. Normality of the distribution of the continuous variables was evaluated using the Kolmogorov–Smirnov test. All other continuous values except QTc, weight, and body surface area (BSA) did not have normal distribution. The Mann–Whitney U test was used to compare these parameters. Correlation analyses were performed using Pearson’s correlation test. All *p*-values were two-tailed, and values <0.05 were indicated as statistically significant.

## 3. Results

**Figure 2:**
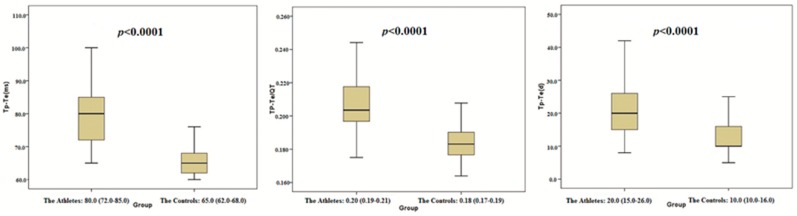
Comparison of Tp-Te, Tp-Te/QT and Tp-Te (d) between the athletes and the controls.

The present study included 565 healthy participants. Of these, 226 (female: 28) were athletes and 339 (female: 45) were healthy controls. We observed the Tp-Te interval, Tp-Te/QT ratio, Tp-Te/corrected QT (QTc) ratio, and Tp-Te(d) (TDR indexes) as well as the PWT, interventricular septal thickness (IVS), LVEDD, LV mass, LV mass index, LVEDV, and LVEDVI to be significantly increased in the athletes as compared with the control group (Table 1 and Figure 2). Additionally, BMI, BSA, EF, and FS were significantly higher in the control group than in the athlete group (Table 1). However, there were no statistically significant differences between the athlete group and the control group in terms of RWT, age, and gender (Table 1).

**Figure 3:**
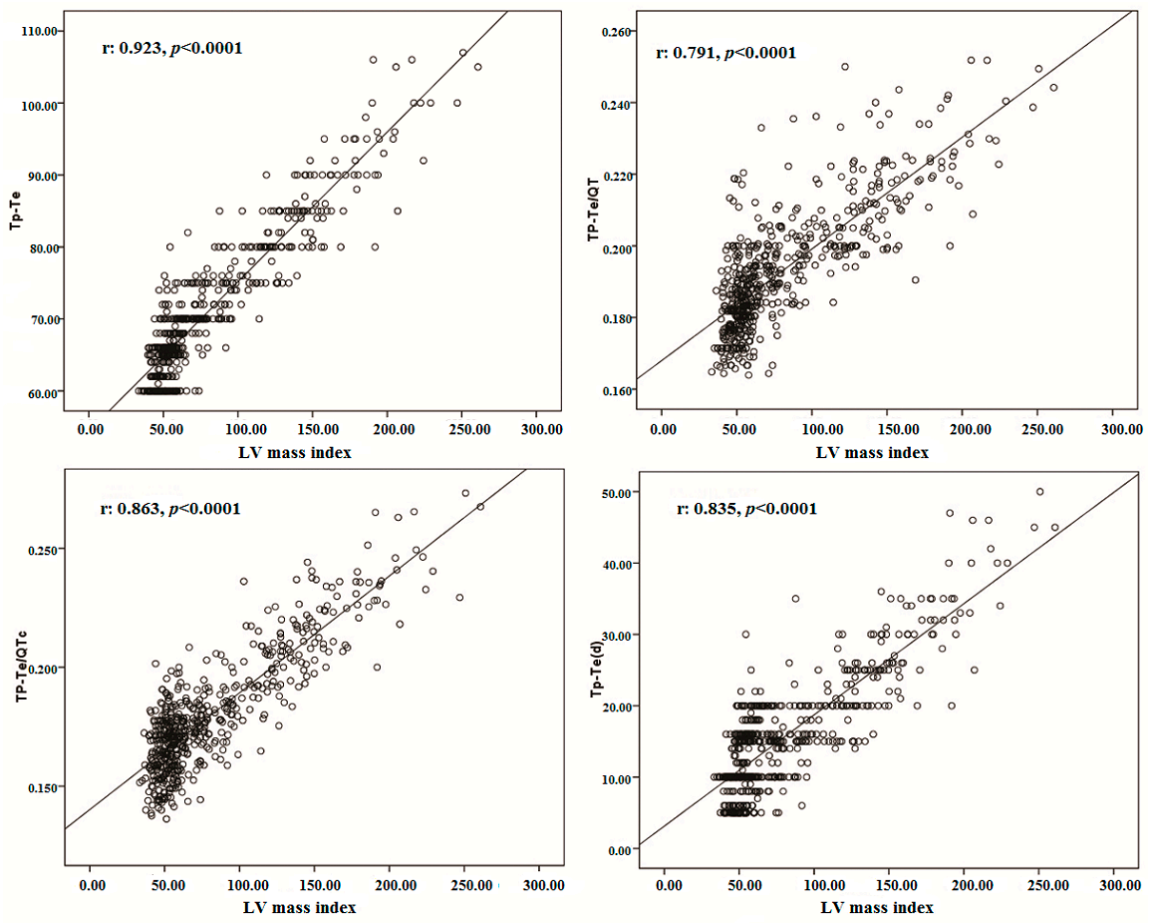
The correlations between Tp-Te, Tp-Te/QT, Tp-Te/QTc, Tp-Te (d) and LV mass index.

Correlation analyses revealed that the TDR indexes were significantly correlated with PWT, IVS, RWT, LVEDD, LV mass, LV mass index, LVEDV, and LVEDVI (Table 2 and Figure 3).

Although a strong positive correlation was detected between training durations–as years and PWT, IVS, LVEDD, LV mass, LV mass index, LVEDV, and LVEDVI, there was a weak positive correlation between training durations–as years and RWT. However, a weak but significant correlation existed between weekly training durations and PWT, IVS, RWT, LV mass, and LV mass index (Table 3). On the other hand, there were no significant differences between weekly training durations and LVEDD, LVEDV, and LVEDVI (Table 3). Interestingly, there was also a strong positive correlation between training durations–as years and TDR indexes. However, a weak significant correlation was found between weekly training durations and TDR indexes (Table 3).

In the athlete group, Tp-Te interval, QTmax, Tp-Te/QT ratio, Tp-Te/QTc ratio, and Tp-Te(d) decreased linearly with an increase in HR. However, despite changes in HR, the Tp-Te/QT ratio values remained constant in the control group. QTc increased linearly with an increase in HR both in the athlete group and in the control group (Table 4).

## 4. Discussion

The present study showed that the Tp-Te interval, Tp-Te/QT, Tp-Te/QTc ratio, and Tp-Te(d) were prolonged in well-trained participants compared with those of the nonathletes (Table 1, Figure 1). We observed that TDR indexes have a strong and significant correlation with PWT, IVS, LVEDD, LVEDV, LVEDVI, LV mass, and LV mass index (Table 2 and Figure 2). On the other hand, there was a positive but weak correlation between RWT and the TDR indexes (Table 2). These findings showed that a total elevation in LV dimension and LV mass is more strongly correlated with ventricular repolarization heterogeneity than its configuration (eccentric hypertrophy or concentric hypertrophy).

Intense exercise may cause heart remodeling to compensate for increases in blood pressure or volume by increasing muscle mass. Cardiac changes do not involve only the left ventricle, but all heart chambers. Physiological cardiac modelling in athletes is associated with normal or enhanced cardiac function. However, recent studies have documented decrements in left ventricular function during intense exercise and the release of cardiac markers of necrosis in athletes’ blood of uncertain significance. Furthermore, it is suggested that cardiac remodeling may predispose athletes to heart disease and results in electrical remodeling, which is responsible for arrhythmias [21,22,23,24]. Consistent with the aforesaid studies, we found a strong positive correlation between training durations-as years and TDR indexes. However, there was a weak correlation between weekly training durations and the same indexes (Table 3). This situation may be explained by the fact that intense and recurrent exercise can cause significant structural heart changes in the long run. In parallel, significant structural heart changes may result in apparent changes in ventricular repolarization homogeneity.

Sudden death includes the deaths that occur within 1 to 6 h after the symptoms appear. In sports-related sudden deaths, deaths often appear within 30 s to 6 h following training or during a competition [25]. The risk of sudden death is 2.5 times greater in athletes than in the normal population. This fact may imply that sports *facilitate* death in such individuals [26]. Nearly 95% of the sudden deaths in athletes are heart-related, and hypertrophic, cardiomyopathic, and malignant ventricular arrhythmia rank among the top conditions that stem from the cardiovascular system [1,25,26,27]. Left ventricle hypertrophy in athletes develops as a response to the pressure or volume loading of the ventricle muscle. “Disorganization” of myocardia fibres is what causes harm in hypertrophic cardiomyopathy. Since the repolarization in hypertrophic myocardial tissue is not homogenous, the risk of lethal arrhythmia and sudden death increases [28]. Distinguishing left ventricle hypertrophy, which appears as a physiological response to exercise in athletes, from hypertrophic cardiomyopathy may be difficult in some cases. In both conditions, a symmetrical increase is observed in the left ventricle wall thickness together with an expansion in left ventricle void diameters without malformation. Normal left ventricle diastolic filling in the left ventricle hypertrophy appears as a result of regular physical exercise. While a septum thickness of less than 13 mm suggests physiological hypertrophy, its being more than 16 mm is in favour of hypertrophic cardiomyopathy [29]. However, the diagnosis of the cases that fall between these numbers may be difficult. This may also make it difficult to determine whether the patient has the risk of ventricular arrhythmia and whether there is the risk of sudden death. In this context, the TDR parameters that can reflect the repolarization heterogeneity of the ventricles that will be measured with 12-derivation electrocardiography may provide us with valuable data in evaluating whether an athletic heart may be prone to ventricular arrhythmia.

Significant differences exist in the action potentials of endocardial, epicardial, and midmyocardial (M) cells that comprise the ventricular myocardium. Relative differences in the time course of repolarization of these 3 cell types is referred to as TDR [30]. The earliest completion of repolarization occurs in the epicardial cells. The peak of the T wave represents the end of the epicardial action potential, and the end of the T wave represents the end of the midmyocardial action potential. Therefore, the Tp-e interval is a reflection of TDR [31]. Additionally, it was reported that there is a strong correlation among Tp-Te(d), sudden death, and ventricular arrhythmias [9]. Gupta et al. suggested that the Tp-Te/ QT ratio may serve as an accurate index for the dispersion of ventricular repolarization due to its independence of alterations in heart rate [10].

Regular, intense sport training induces an increase in the cardiac myocyte length and capacitance [4]. Following cardiac hypertrophy causes action potential duration prolongation of midmyocardial and subepicardial myocytes. Prolongation of action potential duration (APD) is considered to be the electrical response to ventricular hypertrophy. Variations in channel expression highlight the regional effects of hypertrophy in APD. In these regions, the repolarization phases are prolonged, which leads to transmyocardial heterogeneity and, consequently, to malignant arrhythmias [4]. Intensive endurance training may cause fibrous tissue formation in the ventricular myocardium. This may trigger a pathological process for the ventricular arrhythmias as well [32].

Several studies reported that LV dilatation and elevated LV mass may be useful indexes to further risk-stratify for ventricular arrhythmias and sudden cardiac death [28,33,34]. Consistent with the above studies, in the present study, we found that intense exercise causes changes in cardiac structure as assessed through echocardiography and electrocardiography (Table 1, Figure 3). We observed an increment in LV dimensions, LV wall thickness, LV mass, and LV mass index in the athlete group. These findings were strongly associated with elevated Tp-Te interval and Tp-Te/QT and Tp-Te(d) values, which may suggest an increased risk of ventricular arrhythmia. Finally, the present data imply that recurrent intense exercise may cause an increase in the heterogeneity of ventricular repolarization via ventricular structural remodeling. Regular and repetitive heavy exercises cause increases in cardiac cavity diameters, LV wall thickness, and LV mass in athletic hearts that do not have any systemic disease or structural heart disease. In addition, they cause the TDR measured with ECG to increase. In other words, the increasing LV mass and LV mass indexes leave a great mark on surface ECG even when the athlete has no other health concern.

The significant decrease in HR in the athlete group versus the control group is due to the dominant parasympathetic influence occurring during the resting period as a result of regular training [35]. We found the Tp-Te/QT ratio to be correlated with HR negatively in the athletes, while there was no correlation between HR and the Tp-Te/QT ratio in the controls. This suggests that the QTapex (from the onset of the Qwave to the apex of the T wave) interval was more prolonged than the Tp-Te interval in the athletes. The dominant parasympathetic influence may be producing this result as well. Finally, the significant decrease in EF and FS in the athlete group may be caused by elevated LVEDD and LVEDV in addition to the dominant parasympathetic influence.

The present study may contribute to pathophysiological mechanisms of the increased prevalence of ventricular arrhythmias and cardiovascular mortality risk by indicating increased ventricular repolarization heterogeneity in athletes. The increased frequency of ventricular arrhythmia and sudden cardiac death may be explained with prolonged transmural dispersion in these individuals.

## Figures and Tables

**Table 1:** Intergroup comparison of demographical and laboratory data.

	Athlete Group *n*= 226	Control Group *n*= 339	*p*
Age (Year)	25 (21–30)	25 (20–33)	0.63
Gender (Male/Female)	198/28	294/45	0.76
Tp-Te (ms)	80.0 (72.0–85.0)	65.0 (62.0–68.0)	<0.0001
QTmax (ms)	381.0 (369.0–402.0)	356.0 (347.0–365.0)	<0.0001
QTc (ms)	401.29 ± 17.47	393.34 ± 23.23	<0.0001 ^#^
Tp-Te/QT ratio	0.20 (0.19–0.21)	0.18 (0.17–0.19)	<0.0001
Tp-Te/QTc ratio	0.20 (0.18–0.21)	0.16 (0.15–0.17)	<0.0001
Tp-Te(d) (ms)	20 (15–26)	10 (10–16)	<0.0001
HR	65 (57.75–72)	74 (70–80)	<0.0001
PW (mm)	10.0 (9.0–11.0)	8.0 (7.0–8.0)	<0.0001
IVS (mm)	11.0 (10.0–12.0)	8.0 (7.0–8.0)	<0.0001
LVEDD (mm)	54.0 (48.0–58.0)	42.0 (40.0–44.0)	<0.0001
LVESD (mm)	39.0 (34.75–43.0)	27.0 (25.0–29.0)	<0.0001
EF%	54.0 (50.0–58.0)	66.13 (63.26–69.41)	<0.0001
FS%	28.0 (26.0–30.0)	36.1 (34.1–38.6)	<0.0001
RWT%	37.5 (34.5–40.0)	37.0 (34–40)	0.83
LVEDV (mL)	141.31 (107.52–166.56)	78.58 (70.0–87.69)	<0.0001
LVEDVI (mL/m^2^)	75.43 (62.78–87.81)	42 (37.68–46.72)	<0.0001
Length (m)	1.78 (1.75–1.82)	1.74 (1.70–1.76)	<0.0001
Weight (kg)	70.78 ± 9.02	75.65 ± 9.78	<0.0001 ^#^
BMI (kg/m^2^)	21.97 (21.22–22.87)	25.42 (23.18–27.11)	<0.0001
BSA (m^2^)	1.87 ± 0.16	1.90 ± 0.14	0.02 ^#^
LV mass (g)	220.59 (158.82–280.42)	100.60 (89.37–113.63)	<0.0001
LV mass index (g/m^2^)	118.74 (87.94–148.26)	53.22 (47.54–58.88)	<0.0001
SBP (mmHg)	110.0 (100.0–120.0)	115.0 (110.0–120.0)	<0.0001
DBP (mmHg)	70.0 (65.0–70.0)	70.0 (70.0–80.0)	<0.0001

BMI: Body Mass Index, BSA: Body Surface Area, HR: Heart rate, IVS: Interventricular septum, LV: Left ventricle, LVEDD: Left ventricular end-diastolic diameter, LVESD: Left ventricular end-systolic diameter, PW: Posterior wall, EF: Ejection fraction, FS: Fractional shortening, RWT: Relative wall thickness, LVEDV: Left ventricular end-diastolic volume, LVEDVI: Left ventricular end-diastolic volume index, QTmax: QT maximum, ms: millisecond, mm: millimeter, QTc: QT corrected, SBP: Systolic blood pressure, DBP: Diastolic blood pressure. ^#^ Normality of the distribution was evaluated by the Kolmogorov–Smirnov test and the Mann–Whitney *U* test applied to compare for continuous variables except from QTc, weight, and body surface area (BSA).

**Table 2:** Pearson correlation analysis between ventricular repolarisation parameters and echocardiographic parameters.

	Tp-Te (ms)		QTmax (ms)		QTc (ms)		TP-Te/QT Ratio		TP-Te/QTc Ratio		Tp-Te(d) (ms)	
	*r*	*p*	*r*	*p*	*r*	*p*	*r*	*p*	*r*	*p*	*r*	*p*
PW (mm)	0.874	<0.0001	0.799	<0.0001	0.152	<0.0001	0.741	<0.0001	0.808	<0.0001	0.809	<0.0001
IVS (mm)	0.883	<0.0001	0.803	<0.0001	0.173	<0.0001	0.755	<0.0001	0.808	<0.0001	0.792	<0.0001
LVEDD (mm)	0.883	<0.0001	0.771	<0.0001	0.106	0.050	0.776	<0.0001	0.835	<0.0001	0.779	<0.0001
LVESD (mm)	0.872	<0.0001	0.762	<0.0001	0.119	0.020	0.768	<0.0001	0.820	<0.0001	0.758	<0.0001
LVEDV (mL)	0.880	<0.0001	0.768	<0.0001	0.095	0.023	0.771	<0.0001	0.836	<0.0001	0.786	<0.0001
LVEDVI (mL/m^2^)	0.872	<0.0001	0.765	<0.0001	0.114	0.007	0.760	<0.0001	0.820	<0.0001	0.769	<0.0001
RWT%	0.237	<0.0001	0.268	<0.0001	0.122	0.004	0.164	<0.0001	0.186	<0.0001	0.271	<0.0001
LV mass (g)	0.926	<0.0001	0.821	<0.0001	0.117	0.010	0.798	<0.0001	0.872	<0.0001	0.844	<0.0001
LV mass index (g/m^2^)	0.923	<0.0001	0.823	<0.0001	0.132	0.004	0.791	<0.0001	0.863	<0.0001	0.835	<0.0001

IVS: Interventricular septal thickness. LV mass: Left ventricular mass. LVEDD: Left ventricular end-diastolic diameter. PW: Posterior wall thickness.

**Table 3:** Pearson correlation analysis between training durations, TDR indexes, and some echocardiographic parameters.

	Weekly Training Durations		Training Durations–As Years	
	*r*	*p*	*r*	*p*
PW (mm)	0.147	0.02	0.652	<0.0001
IVS (mm)	0.211	0.001	0.625	<0.0001
LVEDD (mm)	0.036	0.06	0.710	<0.0001
LV mass (g)	0.132	0.04	0.732	<0.0001
LV mass index (g/m^2^)	0.144	0.03	0.723	<0.0001
RWT%	0.190	0.004	0.225	0.001
LVEDV (mL)	0.050	0.46	0.702	<0.0001
LVEDVI (mL/m^2^)	0.051	0.44	0.700	<0.0001
Tp-Te (ms)	0.214	0.001	0.712	<0.0001
TP-Te/QT ratio	0.219	0.001	0.555	<0.0001
TP-Te/QTc ratio	0.159	0.017	0.651	<0.0001
Tp-Te (d) (ms)	0.202	0.002	0.668	<0.0001

IVS: Interventricular septal thickness, LV mass: Left ventricular mass, LVEDD: Left ventricular end-diastolic diameter, PW: Posterior wall thickness.

**Table 4:** Pearson correlation analysis between HR and some electrocardiographic repolarization measurements in the athletes and the controls.

	The Athletes		The Controls	
	*r*	*p*	*r*	*p*
Tp-Te (ms)	−0.659	<0.0001	−0.166	0.002
QTmax (ms)	−0.776	<0.0001	−0.366	<0.0001
QTc (ms)	0.652	<0.0001	0.735	<0.0001
Tp-Te/QT ratio	−0.407	<0.0001	0.076	0.16
Tp-Te/QTc ratio	−0.817	<0.0001	−0.667	<0.0001
Tp-Te(d) (ms)	−0.620	<0.0001	−0.147	<0.0001
